# Sleep Respiratory Disorders in Children and Adolescents with Cystic Fibrosis and Primary Ciliary Dyskinesia

**DOI:** 10.3390/children10101707

**Published:** 2023-10-20

**Authors:** Maria Papale, Sara Manti, Santiago Presti, Federico Mollica, Giuseppe F. Parisi, Salvatore Leonardi

**Affiliations:** 1 Department of Clinical and Experimental Medicine, University of Catania, 95124 Catania, Italy; m.papale@policlinico.unict.it (M.P.); gf.parisi@policlinico.unict.it (G.F.P.); leonardi@unict.it (S.L.); 2 Pediatric Unit, Department of Human and Pediatric Pulmonology “Gaetano Barresi”, AOUP G. Martino, University of Messina, 98122 Messina, Italy; 3 Department of Pediatric Pulmonology and Allergology, Sophia Children’s Hospital, 3015 CN Rotterdam, The Netherlands; 4 Department of Radiology and Nuclear Medicine, Erasmus Medical Center, 3015 GD Rotterdam, The Netherlands

**Keywords:** cystic fibrosis, primary ciliary dyskinesia, sleep, sleep respiratory disorders, sleep disorder breathing

## Abstract

Cystic fibrosis (CF) and primary ciliary dyskinesia (PCD) are genetic respiratory diseases featured by chronic upper and lower airway inflammation and infection, mainly due to impaired mucociliary clearance due to genetic mutations. Sleep is crucial to healthy children’s normal physical and psychological development and has an important value in chronic respiratory diseases. Impaired sleep quality, such as sleep deprivation or insufficient sleep during the night, and sleep respiratory disorders (SRDs) are common in 5% to 30% of the general population. Sleep disruption leads to attention deficits, daytime sleepiness, fatigue and mood disorders and correlates to a worsened quality of life. Furthermore, sleep respiratory disorders (SRSs) are under-recognized comorbidities in CF and PCD patients. SRSs include a spectrum of symptoms ranging from primary snoring through upper airway resistance to obstructive sleep apnea (OSA), nocturnal hypoventilation and hypoxemia occurring in people with moderate to severe lung disease and damaging the disease-related outcomes and quality of life. Effective screening during sleep with polysomnography is very important for the timely initiation of efficacious treatments and to prevent worsened respiratory, metabolic and cardiovascular outcomes. However, the impact of SRDs on health and quality of life is still underinvestigated.

## 1. Introduction

Cystic fibrosis (CF) and primary ciliary dyskinesia (PCD) are chronic lung diseases characterized by chronic upper and lower airway inflammation and infection as a result of impaired mucociliary clearance [1].

PCD is a genetically caused disorder of cilia ultrastructure and function, featured by situs viscerum inversus in 50% of patients and impaired mucociliary clearance, resulting in recurrent and chronic infections. The prevalence of PCD ranges from 1:10,000 to 1:20,000 live-born newborns. However, due to different methods adopted for diagnosing PCD, its prevalence shows large variations, ranging from 1:2200 to 1:40,000, with the highest prevalence in Denmark, Switzerland and Cyprus [2]. The main lower airway outcomes are represented by bronchiectasis, asthma exacerbation and recurrent pneumonia; however, in addition, upper airway inflammation and infections such as rhinosinusitis or otitis media are frequently involved, occurring in newborns at a high percentage [2].

CF is a multisystemic disease due to mutations in the cystic fibrosis trans-membrane conductance regulator (CFTR) gene, and it mostly affects the respiratory system but also the pancreas, liver, kidneys and intestine. The incidence of CF is estimated at 1/2500 live white newborns in Europe. However, data from newborn screening programs for CF showed that the incidence was lower than in the past. To date, the incidence of CF is estimated between 1/3000 and 1/6000, which corresponds to carrier rates of 1/28 and 1/40, respectively [3].

CF patients develop chronic pulmonary inflammation and infection with hypersecretion of thickened mucus, which causes airway obstruction, respiratory exacerbation, bronchiectasis and lung failure [3].

Sleep disorders significantly impact physical and cognitive development, mood and quality of life in childhood. Poor sleep induces the production of elevated serum levels of inflammatory markers and increases the tendency toward airway inflammation or infections. In the pediatric population, sleep respiratory disorders (SRDs) include a spectrum of symptoms from primary snoring and upper airway resistance to obstructive sleep apnea syndrome (OSAS). These sleep abnormalities occur even in healthy children, but they may have severe effects in patients with chronic lung disease [4].

CF and PCD patients may develop impaired oxidation and gas exchange during sleep due to bronchial-thickened secretions with lower airway collapse and, eventually, a dysfunction of the central nervous system respiratory center in CF or PCD disease [5].

It has been reported that children with nocturnal desaturations and SRD have a high frequency of lower airway exacerbations such as pneumonia. Nocturnal desaturations contribute to both pulmonary morbidities in CF or PCD patients [6,7]. Furthermore, severe nocturnal airflow limitation in both diseases may contribute to hypoxemia and hypercapnia as a result of impaired gas exchanges. In addition, common disease-related symptoms such as gastroesophageal reflux or musculoskeletal pain lead to recurrent episodes of nocturnal cough and contribute to disturbed sleep architecture and reduced sleep time and efficiency, resulting in daytime sleepiness [8].

The relationship between SRD, sleep quality and sleep quality of life in both diseases has been well documented. Reduced sleep quality of life may be due primarily to lung disease or daily medications that may interfere with sleep [9]. Early assessment and treatment of SRD contribute to a better prognosis and improved clinical care in CF and PCD patients. However, the impact of SRD on health and quality of life is still underinvestigated and not routinely evaluated, both in CF and PCD patients. Accordingly, a shared diagnostic and therapeutic approach in SRDs in these clusters of patients is lacking.

This paper aims to define what is known about SRDs in pediatric patients with CF and PCD and to investigate their implications in clinical practice. Secondly, it highlights new evidence regarding the impact of SRDs on disease-specific outcomes such as pulmonary function.

## 2. Materials and Methods

We searched the PubMed database to conduct this narrative review. We included articles in the English language, and no time limit was adopted. The search strategy was performed using the following keywords: (1) sleep disorder breathing; (2) sleep respiratory disorders; (3) cystic fibrosis; (4) primary ciliary diskynesia; (5) nocturnal hypoxemia; (6) nocturnal ventilation. References of included guidelines were searched to identify any other relevant documents for inclusion.

Exclusion criteria were as follows: case reports, clinical trials, meta-analyses or systematic reviews, and clinical practice guidelines not focusing on sleep disorders in CF and PCD populations. Duplicate documents and papers not relevant to the research question and outcomes, opinion pieces and wrong population/setting/intervention were also excluded.

We performed data extraction using standard templates.

**Figure 1:**
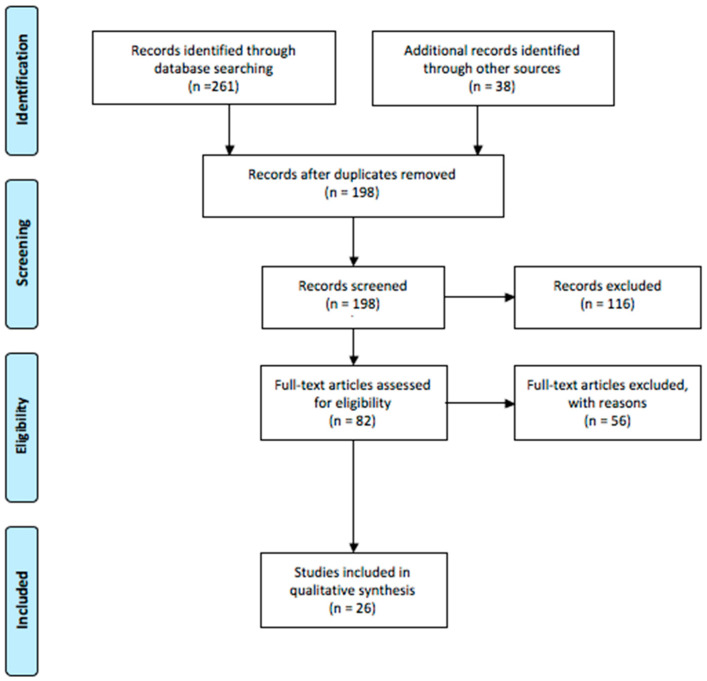
Flow chart of the literature research for two independent reviewers.

A flow chart of the literature research is reported in Figure 1.

## 3. Results

### 3.1. SRDs in CF Patients

Cystic fibrosis (CF) is a life-limiting multisystemic genetic disorder that especially affects pulmonary function, characterized by thickened secretions, resulting in an abnormality of chloride movement [10]. To date, the main aspects responsible for CF-related multisystemic disease have been considered mutations in the cystic fibrosis transmembrane conductance regulators, that is, expressed in the airways. Clinically, CF patients show chronic pulmonary inflammation and infection with hypersecretion of thickened mucus, causing airway obstruction, respiratory exacerbations, bronchiectasis and lung failure [10,11,12].

Most commonly, respiratory failure occurs from cor pulmonale as a result of pulmonary hypertension. It has been demonstrated that chronic nocturnal hypoxia and hypercapnia may contribute to the development of pulmonary hypertension and right ventricular failure. Only lung transplantation may improve the quality of life in CF patients affected by lung and cardiac failure. Several studies have described the relationship between SRD or nocturnal hypoxia and hypercapnia and pulmonary hypertension and cor pulmonale. Early treatment in pediatric or adolescent age influences the outcome and survival [13].

Nocturnal oxyhemoglobin desaturation and low resting daytime pulse oximetry have been shown in patients affected by moderate to severe lung disease. The relationship between SRD and the onset of daytime respiratory failure has been a crucial point of clinical research [14].

Several studies have considered potential daytime predictors of SRDs and nocturnal desaturations in patients with CF [15].

Versteegh et al. showed that resting daytime pulse oximetry is the most important predictor of sleep-related hypoxemia in CF children and adolescents, with a 93.8% value. However, they found that nocturnal desaturation occurred primarily in patients with a forced expiratory value during the first second (FEV1) of less than 65%. However, spirometry did not discriminate the power of resting daytime pulse oximetry to predict nocturnal desaturation. In addition, it has been demonstrated that FEV1 is weakly related to the total sleep time pulse oximetry <90% [16].

Recently, the Lung Clearance Index (LCI), reflecting the overall degree of ventilation inhomogeneity, obtained from a multiple-breath washout of an inert gas marker, has been widely used in CF and PCD children as it studies the distal airways and detects lung damage more accurately than spirometry [17]. It has been shown that LCI has a high effectiveness in predicting sleep disorder breathing, especially nocturnal hypoxemia, in stable CF patients compared with standard parameters of lung function variables, such as FEV1 [18].

The prevalence of OSAS in CF pediatric patients has been reported to be 5–7%. An observational study reported the presence of OSAS in CF children, according to the American Academy of Sleep Medicine definition, as Apnea Hypopnea Index (AHI) > 1/hour. Metabolic factors are considered potential risk factors for CF children and, likewise, in adults developing sleep-disordered breathing [19].

To date, modulators have paradoxically increased rates of obesity and OSAS in CF patients.

Other studies have reported the association between lung function and nocturnal oximetry and described their correlation with measures of severity of lung disease, including airway obstruction and lung hyperinflation. In these studies, nutritional status and respiratory muscle strength did not correlate with nocturnal hypoxemia in the CF population [20].

Braggion et al. studied the relationship between resting oxyhemoglobin pulse oximetry and nocturnal desaturation severity in CF patients with severe lung disease. They found that the airway obstruction degree could not predict the presence of desaturation or its severity [21].

The authors investigated the presence of sleep desaturation and exercise desaturation in 21 adolescents with CF. They concluded that CF patients with normal daytime pulse oximetry values desaturate more during the night than during exercise. There was no relationship between sleep-related hypoxemia or hypercapnia or during exercise and nutritional status, as measured via body mass index. It was found that nocturnal desaturations were uncommon in CF patients with milder lung disease and lung function values of FEV1 higher than 65% of that predicted with spirometry [22]. Millross et al. clinically defined nocturnal significant nocturnal hypoxemia as an SpO_2_ 90% higher than 5% throughout the night and found that morning PaCO_2_ or evening PaO_2_ represented the most predictive factors of sleep-related oxygenation in CF patients from moderate to severe lung disease from NREM to REM sleep [23].

Furthermore, in CF patients, it has been demonstrated that cough significantly impacts sleep disruption. It is very important to start preventive and appropriate therapy after more nocturnal investigations [24].

However, CF patients have periodic acute pulmonary exacerbations affecting lung function, but the relationship between pulmonary exacerbations and SRDs is not clearly reported. Allen et al. studied 45 children with pulmonary exacerbation at the beginning and the end of antibiotic treatment. They showed a daytime saturation lower than previous levels and, above all, a significant impact on nocturnal oxyhemoglobin saturations and worsened sleep architecture [25].

Villa et al. studied nighttime CF children with mild respiratory symptoms and described sleep-related hypoxia, higher apnea–hypopnea indices and lower REM sleep time than asymptomatic patients. They also showed that infants who develop airway inflammation during the first months of life have a higher risk of desaturation during sleep [26].

Another preliminary study described that sleep fragmentation in CF disease could be linked to chronic infective and inflammatory processes, but future studies are needed to better define this possible association [27].

The worsening gas exchange during sleep on daytime activities and the disease’s prognosis have already been clarified, and it would be a useful therapeutic goal toward modifying quality of life and better prognosis in this cluster of patients. Low-flow nocturnal oxygen therapy in CF patients improves oxyhemoglobin saturation during the night, but there are few data on longtime improvements in daytime functions or prognosis [28].

Spier et al. studied, during sleep, patients with CF suffering from moderate to severe lung disease, randomly assigning low-flow oxygen and room air. They found no differences between two groups in sleep quality and parameters of sleep breathing above the number of arousal events [29].

Furthermore, Gozal et al. found that the use of oxygen therapy during sleep in patients with CF with moderate to severe lung disease improved oximetry in all sleep stages studied using polysomnography, albeit accompanying rises in CO_2_ around 5 to 7 mmHg during REM and NREM sleep. This may require some form of awakening or overnight CO_2_ measurement to be predicted prospectively from clinical, laboratory or pulmonary function parameters [30].

In a randomized trial, Ramos et al. studied patients with CF and severe lung disease by comparing room air and long-term nocturnal-flow oxygen, to determine if oxygen therapy during sleep could decrease morbidity or slow down the progression of lung disease. No benefits from the long-term use of oxygen therapy were found [31].

Moreover, the non-invasive ventilation (NIV) efficacy in managing CF patients with moderate to severe lung disease has been discussed enough. Several reports documented a reduction in dyspnea, improvement in respiratory muscle strength and improvement in the quality of sleep and daily activities using NIV.

Currently, NIV in bilevel ventilatory support is adopted as an airway clearance technique in acute pulmonary exacerbation and as a bridge to transplantation in respiratory failure end-stage pulmonary disease. NIV treatment influences sleep and nocturnal breathing parameters, the ventilation/perfusion ratio and distal airway resistance. Moreover, nocturnal hypoventilation, especially during REM sleep, is responsible for sleep-related desaturation in CF patients with moderate to severe lung disease. Therefore, NIV may preserve stable nocturnal CO_2_ levels with the improvement in gas exchange parameters and reduce the respiratory rate during sleep in these patients. To date, the NIV treatment has also been suggested in stable normocapnic CF patients, resulting in favorable clinical outcomes and improvements in quality of life. Despite the widespread use of nocturnal oxygen and NIV in CF patients, many doubts about the effectiveness of these therapeutic supports in morbidity and survival still remain to be elucidated [32,33].

### 3.2. SRD in PCD Patients

PCD is a genetically caused disorder of cilia ultrastructure and function, featured by situs viscerum inversus in 50% of patients and impaired mucociliary clearance, resulting in recurrent and chronic infections. Upper airway diseases, including rhinosinusitis and nasal polyposis, can occur early and cause obstructive sleep apnea syndrome (OSAS) by increasing upper airway resistance. Lower airway manifestations, such as pneumonia, bronchiectasis and asthma, are frequently detected in PCD patients [32,33].

Likewise, in CF disorder, in patients with PCD, the disease progression may be slower, and it is important to track lung function to establish the correct medical therapy and determine prognosis.

It has been reported that SRDs may occur in PCD patients. Only a few studies showed the presence of SRD in PCD patients. SRD complications, such as pulmonary hypertension, metabolic disorders and behavioral or neurocognitive impairment, have a critical role in the prognosis of disease [34,35].

Furthermore, some PCD patients develop pulmonary bronchiectasis, which, in turn, can cause air-trapping and airflow obstruction. These conditions lead to hypoxemia and/or hypercapnia during sleep with gas exchange abnormalities and lung mechanics.

Generally, parents of children with SRDs may often underestimate nighttime sleep quality and sleep disruption [36].

In order to establish a prognosis and optimize the medical management of PCD patients, it is critical to understand the real impact of SRDs in these patients and identify possible risk factors for SRDs.

There are few studies evaluating the rate of SRDs in PCD patients.

Oktem et al. studied twenty-nine PCD children and adolescents and a healthy control with a physical examination, pulmonary function tests, an ear–nose–throat evaluation, a sleep quality questionnaire and overnight polysomnography. They aimed to investigate the rate of sleep quality and SRDs in patients with PCD and whether these complications may be related to lung disease severity and upper airway inflammation. They found that PCD patients have worsened sleep quality and had a higher rate of SRDs than the control group. Cigarette smoke might be a risk factor for OSAS in these patients [37].

Aside from this, Santamaria et al. simultaneously analyzed the presence of SRDs and sleep quality via polysomnography and the rate of sinonasal or lung disease via pulmonary function tests and computed tomography (CT) in children and adolescents with PCD. They discovered that many PCD patients commonly had OSAS from mild to moderate degrees without correlation with chronic rhinosinusitis severity. Furthermore, nocturnal oxygen saturation values significantly correlate with pulmonary structure abnormalities observed via CT [38].

However, there are several limitations in these studies. First, there are no data about sleep macrostructure and its relationship with respiratory disorders. Second, they included only stable patients and did not compare them with nonstable diseases to determine the potential role of pulmonary exacerbations and the effects of treatment on SRDs [39].

In conclusion, cardiorespiratory nocturnal monitoring is very important during the follow-up of PCD disease, and the treatment and assessment of sleep disorder breathing might contribute to better prognosis and improved clinical care, but further exploration is needed.

Table 1 summarizes the study characteristics.

## 4. Discussion

SRDs in infants, children and adolescents are different entities in both definition and presentation according to the AASM scoring manual. People with chronic pulmonary disease, such as CF or PCD, may be at potential risk of developing sleep-disordered breathing [40,41].

In these conditions, SRDs can potentially be suspected due to daytime sleepiness, daytime oxygen saturation, pulmonary function and nutritional status. In patients with moderate or severe pulmonary disease, increased effort in breathing during sleep can be a sign of nocturnal hypoxemia. Children with CF have frequent occurrences of hypoxemia and hypercapnia during sleep. At the same time, SRDs can also occur in PCD patients [42,43].

It has been shown that SRDs, especially in patients with moderate to severe lung diseases, such as CF or PCD, are associated with behavioral disturbances, cognitive impairment, metabolic consequences and an increased risk of cardiovascular disease. Nighttime cough is a common symptom in both CF and PCD patients and causes recurring awakening from sleep [44]. To date, there are no screening guidelines for SRDs in CF and PCD patients. The gold standard for diagnosis of SRDs is polysomnography, but respiratory polygraphy has been shown to be an attested substitute for well-studied sleep in these patients [45,46]. In fact, daytime measurements of oxygen saturation, lung function as FEV1 or LCI, and arterial blood gas measurements are not useful alone in predicting SRDs [15,16,17,18].

There are no clear guidelines for the treatment of isolated nocturnal hypoxemia in CF or PCD patients.

## 5. Conclusions

Herein, according to the recent literature, we summarized the important impact of SRDs on CF and PCD in the pediatric population (Table 2). SRDs are common in these population clusters; however, they are largely overlooked. The underdiagnosis of SRD results in multiple adverse long-term effects, such as OSA, nocturnal hypoxemia and hypercapnia, and hypoventilation and, lastly, worsening the multi-organ morbidity of CF and PCD. Studies highlight important points when considering sleep in the routine care of CF and PCD children. Sleep should be evaluated routinely in CF and PCD prospective studies confirming the benefits of precocious interventions to improve sleep duration and quality. Moreover, further research is needed to establish the correct approach to SRDs in CF and PCD patients.

## Figures and Tables

**Table 1:** Characteristics of the included studies. SRD: sleep respiratory disorders; CF: cystic fibrosis; PCD: Primary Ciliary Diskynesia; QoL: quality of life; FEV1: Forced Expiratory Volume in the first second.

CF						
Authors	Type of Study	Number of Patients	Mean Age (yrs)	Variable Assessed	Main Findings	References
Cohen-Cymberknoh et al., 2019	Observational	39	11.1	Lung function, sleep disorders and their correlation with QoL	Sleep impairment correlated with disease severity and affected QoL.	[4]
Isaiah et al., 2019	Retrospective case series	41	11.6	Pulmonary function, polysomnographic variables	FEV1 was the best predictor of sleep hypoxemia in children with CF and referred for polysomnography.	[14]
Perin et al., 2012	Prospective	51	25.1 ± 6.7	Cardiac and pulmonary function, and polysomnographic variables	Desaturation was common and not associated with obstructive events during sleep. It can be predicted by awake resting SpO_2_ < 94%	[15]
Papale et al., 2020	Observational	31	17.4 ± 5.2	Lung function and sleep disorders	LCI has a higher effectiveness in predicting nocturnal hypoxemia in stable patients with CF than traditional parameter of lung function such as FEV 1 .	[18]
Spicuzza L. et al.	Case-control	40	6.3 ± 5.6	Polysomnographic variables and sleep quality	Early occurrence of obstructive sleep apnea in children with CF in stable condition.	[19]
Milross et al., 2002	Observational	31	27 ± 8	First-night effect of polysomnographic measurements on nocturnal oxygenation	A single-night with polysomnographic measurements in patients with CF provided information on nocturnal oxygenation and respiratory disturbance.	[23]
De Castro Silva et al., 2009	Prospective	30	12.8	Polysomnographic variables	Desaturation during sleep can be predicted by FEV1 < 64%	[25]
Paranjape et al., 2015	Observational	43	9.6 ± 3.6	Analysis of breathing patterns, gas exchange and polysomnographic variables.	Children with CF demonstrated lower oxyhemoglobin saturation and a higher proportion of inspiratory flow limitation, compared with control group	[27]
**PCD**						
**Authors**	**Type of study**	**Number of patients**	**Mean age (yrs)**	**Variable assessed**	**Main findings**	**References**
Cohen-Cymberknoh et al., 2019	Observational	39	11.1	Lung function, sleep disorders and their correlation with QoL	Sleep impairment correlated with disease severity and affected QoL.	[4]
Oktem et al., 2013	Observational Case control	29	10.9	Sleep quality and sleep disorders	Patients with PCD have decreased sleep quality and higher rate of OSAS compared to controls	[39]
Santamaria et al., 2014	Observational Case control	60	12.3	Lung function, nasal endoscopy and sleep disorders	Nocturnal desaturation was linked with lung function and structure abnormalities	[40]

**Table 2:** Recommendations and practice points for nocturnal monitoring in CF and PCD patients, based on current literature.

Clinical Parameters Predicting of Sleep Respiratory Disorders	Monitoring
Resting SpO_2_ < 94% Lung function with FEV1 <65% predicted Daytime symptoms as excessive daytime sleepiness and headache Worsened quality of life Decreased muscle strength	Continuous monitoring of SpO_2_ during sleep CO_2_ monitoring during sleep or awakening blood gases analysis Nocturnal cardiorespiratory polygraphy and/or polysomnography with associated periodic follow-up

## References

[1] Cohen-Cymberknoh M., Simanovsky N., Hiller N., Hillel A.G., Shoseyov D., Kerem E. (2014). Differences in disease expression between primary ciliary dyskinesia and cystic fibrosis with and without pancreatic insufficiency. Chest.

[2] Shapiro A.J., Davis S.D., Polineni D., Manion M., Rosenfeld M., Dell S.D., Chilvers M.A., Ferkol T.W., Zariwala M.A., Sagel S.D. (2018). Diagnosis of Primary Ciliary Dyskinesia. An Official American Thoracic Society Clinical Practice Guideline. Am. J. Respir. Crit. Care Med..

[3] Farrell P.M., White T.B., Ren C.L., Hempstead S.E., Accurso F., Derichs N., Howenstine M., McColley S.A., Rock M., Rosenfeld M. (2017). Diagnosis of Cystic Fibrosis: Consensus Guidelines from the Cystic Fibrosis Foundation. J. Pediatr..

[4] Cohen-Cymberknoh M., Atia O., Gileles-Hillel A., Kerem E., Reiter J. (2019). Sleep disorders in patients with primary ciliary dyskinesia, cystic fibrosis with and without pancreatic insufficiency. Respir. Med..

[5] Naqvi S.K., Sotelo C., Murry L., Simakajornboon N. (2008). Sleep architecture in children and adolescents with cystic fibrosis and the association with severity of lung disease. Sleep Breath..

[6] Amin R., Bean J., Burklow K., Jeffries J. (2005). The relationship between sleep disturbance and pulmonary function in stable pediatric cystic fibrosis patients. Chest.

[7] Milross M.A., Piper A.J., Norman M., Dobbin C.J., Grunstein R.R., E Sullivan C., Bye P.T. (2002). Subjective sleep quality in cystic fibrosis. Sleep Med..

[8] Vandeleur M., Walter L.M., Armstrong D.S., Robinson P., Nixon G.M., Horne R.S. (2018). Quality of life and mood in children with cystic fibrosis: Associations with sleep quality. J. Cyst. Fibros..

[9] Johnson D.A., Billings M.E., Hale L. (2018). Environmental Determinants of Insufficient Sleep and Sleep Disorders: Implications for Population Health. Curr. Epidemiol. Rep..

[10] Abbott J., Elborn J.S., Georgiopoulos A., Goldbeck L., Marshall B., Sabadosa K., Smith B., Quittner A. (2015). Cystic Fibrosis Foundation and European Cystic Fibrosis Society Survey of cystic fibrosis mental health care delivery. J. Cyst. Fibros..

[11] Parisi G.F., Papale M., Tardino L., Nenna R., Midulla F., Leonardi S. (2019). Biomarkers in Pediatric Lung Diseases Including Cystic Fibrosis. Curr. Respir. Med. Rev..

[12] Manti S., Parisi G.F., Papale M., Marseglia G.L., Licari A., Leonardi S. (2022). Type 2 inflammation in cystic fibrosis: New insights. Pediatr Allergy Immunol..

[13] Urquhart D.S., Montgomery H., Jaffé A. (2005). Assessment of hypoxia in children with cystic fibrosis. Arch. Dis. Child..

[14] Isaiah A., Daher A., Sharma P.B. (2019). Predictors of sleep hypoxemia with cystic fibrosis. Pediatr. Pulmonol..

[15] Perin C., Fagondes S.C., Casarotto F.C., Pinotti A.F.F., Barreto S.S.M., Dalcin P.d.T.R. (2012). Sleep findings and predictors of sleep desaturation in adult cystic fibrosis patients. Sleep Breath..

[16] Versteegh F.G., Bogaard J.M., Raatgever J., Stam H., Neijens H., Kerrebijn K. (1990). Relationship between airway obstruction, desaturation during exercise and nocturnal hypoxaemia in cystic fibrosis patients. Eur. Respir. J..

[17] Poncin W., Lebecque P. (2019). L’indice de clairance pulmonaire dans la mucoviscidose [Lung clearance index in cystic fibrosis]. Rev. Mal. Respir..

[18] Papale M., Parisi G.F., Spicuzza L., Licari A., Bongiovanni A., Mulè E., Rotolo N., Manti S., Leonardi S. (2020). Lung clearance index evaluation in detecting nocturnal hypoxemia in cystic fibrosis patients: Toward a new diagnostic tool. Respir. Med..

[19] Spicuzza L., Sciuto C., Leonardi S., La Rosa M. (2012). Early occurrence of obstructive sleep apnea in infants and children with cystic fibrosis. Arch. Pediatr. Adolesc. Med..

[20] American Thoracic Society (1995). Standardization of Spirometry, 1994 Update. Am. J. Respir. Crit. Care Med..

[21] Braggion C., Pradal U., Mastella G. (1992). Hemoglobin desaturation during sleep and daytime in patients with cystic fibrosis and severe airway obstruction. Acta Paediatr..

[22] Silva A.M., Descalço A., Salgueiro M., Pereira L., Barreto C., Bandeira T., Ferreira R. (2016). Respiratory sleep disturbance in children and adolescents with cystic fibrosis. Rev. Port. Pneumol..

[23] Milross M.A., Piper A.J., Norman M., Willson G.N., Grunstein R.R., E Sullivan C., Bye P.T. (2002). Night-to-night variability in sleep in cystic fibrosis. Sleep Med..

[24] Young A., Hassett C. (2002). Treatment of exacerbations of cystic fibrosis improves nocturnal desaturation independent of changes in FEV1. Respirology.

[25] De Castro-Silva C., de Bruin V.M., Cavalcante A.G.M., Bittencourt L.R.A., de Bruin P.F.C. (2009). Nocturnal hypoxia and sleep disturbances in cystic fibrosis. Pediatr. Pulmonol..

[26] Villa M.P., Pagani J., Lucidi V., Palamides S., Ronchetti R. (2001). Nocturnal oximetry in infants with cystic fibrosis. Arch. Dis. Child..

[27] Paranjape S.M., McGinley B.M., Braun A.T., Schneider H. (2015). Polysomnographic Markers in Children with Cystic Fibrosis Lung Disease. Pediatrics.

[28] Van der Giessen L., Loeve M., de Jongste J., Hop W., Tiddens H. (2009). Nocturnal cough in children with stable cystic fibrosis. Pediatr. Pulmonol..

[29] Spier S., Rivlin J., Hughes D., Levison H. (1984). The effect of oxygen on sleep, blood gases, and ventilation in cystic fibrosis. Am. Rev. Respir. Dis..

[30] Gozal D. (1997). Nocturnal ventilatory support in patients with cystic fibrosis: Comparison with supplemental oxygen. Eur. Respir. J..

[31] Ramos R.T., Santana M.A., Almeida P.d.C., Júnior A.d.S.M., Araújo-Filho J.B., Salles C. (2013). Nocturnal hypoxemia in children and adolescents with cystic fibrosis. J. Bras. Pneumol..

[32] Moran F., Bradley J.M., Piper A.J. (2017). Non-invasive ventilation for cystic fibrosis. Cochrane Database Syst. Rev..

[33] Fauroux B., Le Roux E., Ravilly S., Bellis G., Clément A. (2008). Long-term noninvasive ventilation in patients with cystic fibrosis. Respiration.

[34] Knowles M.R., Daniels L.A., Davis S.D., Zariwala M.A., Leigh M.W. (2013). Primary ciliary dyskinesia. Recent advances in diagnostics, genetics, and characterization of clinical disease. Am. J. Respir. Crit. Care Med..

[35] Ullmann N., Santamaria F., Allegorico A., Fainardi V., Borrelli M., Ferraro V.A., Proietti E., Parisi G.F., Romagnoli V., Lucca F. (2023). Primary ciliary dyskinesia: A multicenter survey on clinical practice and patient management in Italy. Pediatr. Pulmonol..

[36] Sommer J.U., Schäfer K., Omran H., Olbrich H., Wallmeier J., Blum A., Hörmann K., Stuck B.A. (2011). ENT manifestations in patients with primary ciliary dyskinesia: Prevalence and significance of otorhinolaryngologic co-morbidities. Eur. Arch. Oto-Rhino-Laryngol..

[37] Maglione M., Bush A., Montella S., Mollica C., Manna A., Esposito A., Santamaria F. (2012). Progression of lung disease in primary ciliary dyskinesia: Is spirometry less accurate than CT?. Pediatr. Pulmonol..

[38] Kieckhefer G.M., Lentz M.J., Tsai S.-Y., Ward T.M. (2009). Parent-child agreement in report of nighttime respiratory symptoms and sleep disruptions and quality. J. Pediatr. Health Care.

[39] Oktem S., Karadag B., Erdem E., Gokdemir Y., Karakoc F., Dagli E., Ersu R. (2013). Sleep disordered breathing in patients with primary ciliary dyskinesia. Pediatr. Pulmonol..

[40] Santamaria F., Esposito M., Montella S., Cantone E., Mollica C., De Stefano S., Mirra V., Carotenuto M. (2014). Sleep disordered breathing and airway disease in primary ciliary dyskinesia. Respirology.

[41] Goldbart A.D., Tal A., Givon-Lavi N., Bar-Ziv J., Dagan R., Greenberg D. (2012). Sleep-disordered breathing is a risk factor for community-acquired alveolar pneumonia in early childhood. Chest.

[42] Goh D.Y., Galster P., Marcus C.L. (2000). Sleep architecture and respiratory disturbances in children with obstructive sleep apnea. Am. J. Respir. Crit. Care Med..

[43] American Academy of Pediatrics (2002). Section on Pediatric Pulmonology, Subcommittee on Obstructive Sleep Apnea Syndrome. Clinical practice guideline: Diagnosis and management of childhood obstructive sleep apnea syndrome. Pediatrics.

[44] Helbich T.H., Heinz-Peer G., Eichler I., Wunderbaldinger P., Götz M., Wojnarowski C., Brasch R.C., Herold C.J. (1999). Cystic fibrosis: CT assessment of lung involvement in children and adults. Radiology.

[45] Bush A., Payne D., Pike S., Jenkins G., Henke M.O., Rubin B.K. (2006). Mucus properties in children with primary ciliary dyskinesia: Comparison with cystic fibrosis. Chest.

[46] Green K., Buchvald F.F., Marthin J.K., Hanel B., Gustafsson P.M., Nielsen K.G. (2012). Ventilation inhomogeneity in children with primary ciliary dyskinesia. Thorax.

